# Genomic analysis reveals the major driving forces of bacterial life in the rhizosphere

**DOI:** 10.1186/gb-2007-8-9-r179

**Published:** 2007-09-04

**Authors:** Miguel A Matilla, Manuel Espinosa-Urgel, José J Rodríguez-Herva, Juan L Ramos, María Isabel Ramos-González

**Affiliations:** 1 Department of Environmental Protection, Estación Experimental de Zaidín, Consejo Superior de Investigaciones Científicas (CSIC), Profesor Albareda 1, Granada 18008, Spain

## Abstract

**Background:**

Mutualistic interactions less well known than those between rhizobia and legumes are commonly found between plants and bacteria, frequently pseudomonads, which colonize roots and adjacent soil areas (the rhizosphere).

**Results:**

A global analysis of *Pseudomonas putida *genes expressed during their interaction with maize roots revealed how a bacterial population adjusts its genetic program to this lifestyle. Differentially expressed genes were identified by comparing rhizosphere-colonizing populations with three distinct controls covering a variety of nutrients, growth phases and life styles (planktonic and sessile). Ninety rhizosphere up-regulated (*rup*) genes, which were induced relative to all three controls, were identified, whereas there was no repressed gene in common between the experiments. Genes involved in amino acid uptake and metabolism of aromatic compounds were preferentially expressed in the rhizosphere, which reflects the availability of particular nutrients in root exudates. The induction of efflux pumps and enzymes for glutathione metabolism indicates that adaptation to adverse conditions and stress (oxidative) response are crucial for bacterial life in this environment. The finding of a GGDEF/EAL domain response regulator among the induced genes suggests a role for the turnover of the secondary messenger c-diGMP in root colonization. Several mutants in *rup *genes showed reduced fitness in competitive root colonization.

**Conclusion:**

Our results show the importance of two selective forces of different nature to colonize the rhizosphere: stress adaptation and availability of particular nutrients. We also identify new traits conferring bacterial survival in this niche and open a way to the characterization of specific signalling and regulatory processes governing the plant-*Pseudomonas *association.

## Abstract 2

A global analysis of *Pseudomonas putida *gene expression performed during the interaction with maize roots revealed how a bacterial population adjusts its genetic program to the specific conditions of this lifestyle.

## Background

The surface of plant roots and the surrounding soil area constitute a complex environment, referred to as the rhizosphere, where microbial activity is high, sustained by the release of nutrients through plant root exudates. This results in a bacterial population density that is one to two orders of magnitude higher than in bulk soil [1,2]. However, the diversity of bacterial species colonizing this habitat is significantly lower than that found in other soil regions [3], suggesting that strong selective forces are at play in the rhizosphere. Part of this selective pressure is likely posed by the plant in the form of specific nutrients, secondary metabolites or signaling molecules in root exudates, and may constitute a means to promote mutualistic relationships with beneficial microorganisms. Although the best known example of such interactions is the endosymbiotic association of rhizobia with legume roots, other less studied instances of mutualism are commonly found between many plant species and rhizosphere-colonizing bacteria with plant growth promoting or disease suppressing activities [4,5]. Studying the gene expression program of a plant-beneficial bacterial population in the rhizosphere may shed light on the mechanisms underlying the establishment of mutualistic interactions between prokaryotic and eukaryotic organisms. It should allow us to explore in detail the determinants required by bacteria to adapt to and colonize this habitat, and provide a better understanding of sessile bacterial growth (that is, microcolony and biofilm formation) in association with biotic surfaces.

Previous efforts aimed at dissecting the genetic program of beneficial *Pseudomonas *in their association with plants have relied on *in vivo *expression technology. These studies provided useful yet limited information, since genome coverage was estimated to be 10-17% [6,7]. Nevertheless, *in vivo *expression technology can be effective to identify genes whose expression patterns would render them less apparent in microarray experiments, and provides a view at the single cell rather than the population level. Transcriptional profiling of *P. aeruginosa *after adding root exudates to laboratory growth medium has also been recently reported [8]. In our work we have performed a realistic approach, analyzing bacterial cells from the rhizosphere so that conditions characteristic of this situation, in particular the association of bacterial cells with the plant root surface and milieu and the continuous supply of exudates, are taken into account. Plants are not passive guests in this interaction, as can be deduced from the modifications observed in their gene expression patterns, not only locally in the root but also in the aerial parts. This systemic response was observed after infection of rhizobacteria-colonized *Arabidopsis *by phytopathogenic agents in comparison to non-colonized plants [9]. Overall, this work answers part of the increasingly recognized necessity of applying genomewide approaches to unveil microbial functioning in plant-bacterial interactions [10].

## Results and discussion

### Analysis of the *Pseudomonas putida *genetic program in the rhizosphere

To investigate how *Pseudomonas *populations readjust their genetic program upon establishment of a mutualistic interaction with plants, we have performed a genome-wide analysis of gene expression of the root-colonizing bacterium *Pseudomonas putida *KT2440 in the rhizosphere of corn (*Zea mays *var. Girona), using microarrays (ArrayExpress repository for microarray data, accession number A-MEXP-949). Among other relevant characteristics, this strain is an excellent root colonizer of plants of interest in agriculture [7] and activates induced systemic resistance against certain plant pathogens (Matilla *et al*., in preparation). Different experiments were designed in order to obtain as broad a picture as possible, comparing rhizosphere populations with three alternative controls: planktonic cells growing exponentially in rich medium (LB medium); planktonic cells in stationary phase in LB medium; and sessile populations established in sand microcosms (defined medium), under the same conditions used to grow inoculated corn plants (see Materials and methods). The combination of these diverse growth conditions balances the contribution of parameters such as growth phase, nutrients and life style to any observed changes in gene expression. Unveiling differentially expressed genes common to all the studies would minimize noise and allow us to identify genes with a reliable and specific change in their expression level in the rhizosphere, likely to be important for survival in this environment. RNA samples were obtained from bacterial cells recovered from the rhizosphere six days after inoculation of gnotobiotic seedlings, and from each of the control settings. Microarrays were hybridized with equal amounts of differentially labeled cDNA and examined for up- and down-regulated genes. Data were processed in two separate ways. The first consisted of evaluating every single experiment (consisting of three biological replicas each) independently, followed by the imposition that genes showing significant changes in gene expression did so in the three experiments, each with a different control. The second analysis evaluated these three experiments through a combined examination of the nine microarrays altogether, followed by a *P *value adjustment. Finally, the results from both data treatments were compared.

**Figure 1:**
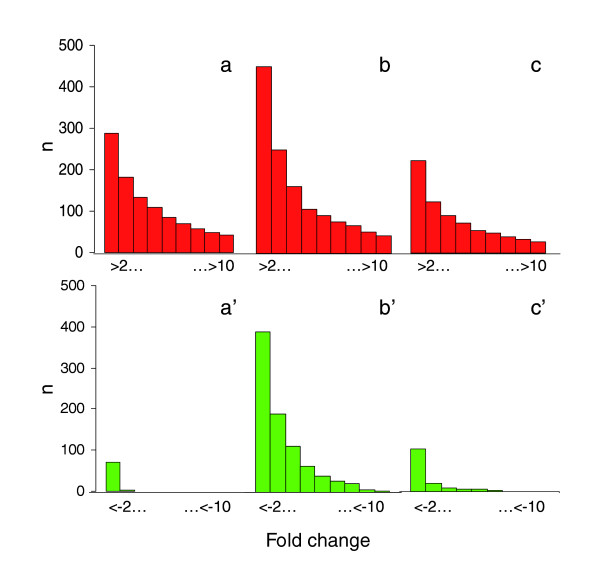
Microarray profiling of bacterial gene expression in the rhizosphere. **(a-c) **Global gene expression of *P. putida *KT2440 was analyzed in the rhizosphere versus that of LB log bacteria (OD_660 _= 0.7) (a, a'), LB stationary phase cells (OD_660 _= 3.3) (b, b'), and sessile bacterial cells incubated in sand microcosms (c, c'). Experimental set up and cDNA preparation are described in detail in Materials and methods. Genes induced (red) and repressed (green), with a *P *value < 0.05 and A >64 were clustered according to their fold change (>2 to >10) and (≤2 to ≤10) and the number is plotted.

**Figure 2:**
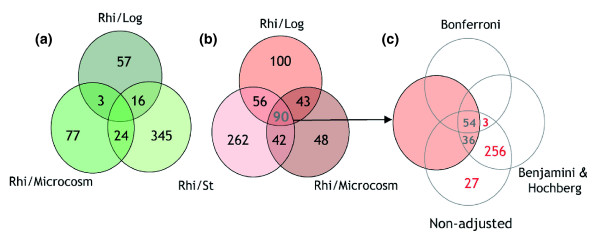
Venn diagrams showing the overlap between differentially expressed genes in the rhizosphere. **(a) **Down-regulated and **(b) **up-regulated genes resulting from the individual analysis of each experiment: rhizosphere versus LB exponentially growing cells (Rhi/Log), rhizosphere versus LB stationary phase cells (Rhi/St), and rhizosphere versus sessile cells in microcosm without plant (Rhi/Microcosm). Common genes were clustered automatically with a freely available informatics tool [48]. **(c) **The result of the combined analysis of the three experiments before and after applying adjustments in the *P *value. Out of the 57 Bonferroni genes, 54 are included in the 90 overlapping *rup *genes.

Two general observations can be highlighted when rhizospheric KT2440 bacteria are compared to their control counterpart by analyzing each experiment individually. The first is that gene activation is more conspicuous than gene repression in the bacterial rhizospheric life style, as reflected by the fact that over 50 genes were induced more than 6-fold in the three experiments (Figure 1). In total, 90 genes appeared consistently up-regulated in the rhizosphere versus all three controls (fold change >2, *P *value < 0.05), and none down-regulated (fold change <-2, *P *value < 0.05) (Figure 2; Additional data file 1). A selection of up-regulated genes is listed in Table 1. The second relevant result was that sessile *P. putida *growing in sand microcosms and stationary phase cells exhibited the most comparable and the most dissimilar gene expression pattern, respectively, with respect to rhizosphere cells (Figure 1). It is worth noting that many genes encoding ribosomal proteins are induced in the rhizosphere after six days of colonization compared to stationary phase (Additional data file 1), indicating the existence of active growth and metabolism, at least in a subpopulation of cells. These results offer a view of *P. putida *life in association with plant roots as a situation where metabolically active bacterial cells grow in a biofilm-resembling state [11], although with their genetic program adjusted to the presence of the plant.

**Table 1:** Rhizosphere up-regulated (*rup*) genes

	Fold change			
	
Locus - TIGR annotation	Log*	St.^†^	Sand^‡^	Combined^§^
**Cytochrome biosynthesis**				
PP0109 - membrane protein putative	*P *> 0.05	10.3	7.9	7.7
PP0110 - *cyoE-1*-protoheme IX farnesyltransferase	13.2	51.5	10.2	-
PP3183 - SCO1/SenC family protein/cytochrome c	2.9	5	3	-
				
**Metabolism**				
PP0326 - *soxG*-sarcosine oxidase gamma subunit	4.8	7.9	5.8	6
PP1403 - *bglX*-periplasmic beta-glucosidase	2.5	2.9	2.5	2.6
PP2694 - aldehyde dehydrogenase family protein	9	8.3	LS	10.2
PP2847 - *ureJ*-urease accessory protein UreJ	22.9	29.6	21.9	24.6
PP3281 - phenylacetic acid degradation protein PaaI putative	6.2	8.1	8.6	7.5
PP3352 - arylsulfatase putative	36.5	17.6	49.4	31.5
PP3746 - *glcE*-glycolate oxidase subunit GlcE	3.6	3.4	3.6	3.5
PP3923 - phosphoglycerate mutase family protein	4.5	4.3	2.8	3.8
PP4588 - nitroreductase family protein	2.6	2.3	3.3	2.7
PP4782 - *thiD*-phosphomethylpyrimidine kinase	8.5	5.8	5.5	6.5
PP5076 - *gltB*-glutamate synthase large subunit	3.4	5.5	2.1	-
PP5197 - *ubiF*-2-octaprenyl-3-methyl-6-methoxy-1,4-benzoquinol hydroxylase	6.9	3.5	5.8	5.2
				
**Secondary metabolism**				
PP3786 - aminotransferase	5.3	4.3	2.1	-
				
**Chemotaxis and motility**				
PP4331 - conserved hypothetical protein	4.9	5.6	4.5	5
PP4359 - *fliL*-flagellar protein FliL	4	4.6	2.8	3.7
PP4391 - *flgB*-flagellar basal-body rod protein FlgB	3	5.2	5.2	4.3
PP4987 - chemotaxis protein putative	6.4	7.3	4.4	5.9
				
**Regulators and sensor proteins**				
PP1066 - sigma-54 dependent response regulator	13	41.9	10.1	17.6
PP3640 - transcriptional regulator AraC family	19.7	28.9	10.3	-
PP4295 - transcriptional regulator TetR family	8.9	8.7	6	7.7
PP4508 - transcriptional regulator AraC family	3.2	2.7	3.2	3
PP0700 - transmembrane sensor putative	21.9	50.4	26.2	30.7
PP2127 - sensor histidine kinase	31.8	14.1	17.3	19.8
PP4959 - sensory box protein/response regulator	14.7	5.1	9.4	8.9
PP5321 - *phoR*-sensory box histidine kinase PhoR	10.5	9.1	9.7	9.8
				
**Stress adaptation and detoxification**				
PP0373 - Pmp3 family protein	8.1	8.4	8	8.1
PP1874 - glutathione peroxidase (GSH_peroxidase)	4.3	7.9	5.4	5.7
PP2376 - *cti*-esterified fatty acid *cis*/*trans *isomerase	2.4	3.6	2.3	-
PP3535 - *ggt-1*-gamma-glutamyltransferase	2.2	2.2	2.1	2.2
				
**ABC transporters**				
PP0196 - ABC transporter ATP-binding protein putative	2.4	6.7	3.7	-
PP2669 - outer membrane protein putative	9.5	11.6	4.7	8
PP3210 - ABC transporter pernease protein	3.4	4.3	3.9	3.8
PP3223 - ABC transporter periplasmic binding protein (dipeptide)	36.9	25.4	66.4	39.5
PP3802 - cation ABC transporter ATP-binding protein putative	13.8	20.1	5.2	-
PP4305 - periplasmic thiosulfate-binding protein	3.2	3.8	2.7	3.2
PP4483 - basic amino acid ABC transporter ATP-binding protein	3.5	4.6	2.6	3.5
				
**Efflux pumps**				
PP0670 - transporter bile acid/Na+ symporter family	5.7	11.1	3.7	-
PP0906 - multidrug efflux RND transporter putative	3.5	8.5	2.6	-
PP1271 - multidrug efflux MFS transporter putative	11.3	25.6	18.1	17.4
PP2817 - *mexC*-multidrug efflux RND membrane fusion protein MexC	3.8	6	2.2	-
PP3583 - RND efflux transporter permease protein	2.7	4.2	3	-
				
**Other transporters**				
PP2385 - *azlC*-branched-chain amino acid transport protein AzlC	4	4.1	3.2	3.8
PP3132 - polysaccharide transporter putative	3.1	3.9	2.6	3.2
PP5297 - amino acid transporter putative (polyamines)	6.6	8.8	6.7	7.3
				
**DNA replication, recombination and repair**				
PP1476 - conserved hypothetical protein	17.3	76.2	29	33.7
PP2565 - helicase putative	5.5	12.6	5.1	-
PP3966 - ISPpu14 transposase Orf1	17.3	11.4	8.1	11.7
				
**Others**				
PP2076 - hypothetical protein	4.4	*P *> 0.05	7.5	5.2
PP2155 - *lolD*-lipoprotein releasing system ATP-binding protein	4	2.4	5.2	-
PP2560 - transport protein HasD putative	23.3	60	9	-
PP3184 - hypothetical protein	6.6	3.9	3.9	4.6

Proteins with predicted general function and hypothetical proteins not mentioned in the text are not included (Additional data file 1). Although the *P. putida *KT2440 genome is sequenced and annotated [49], the locus functions listed in this table were one by one re-confirmed by comparing the amino acid sequences with those in the databases. The complete list of *rup *genes (genes with fold induction >2, *P *value < 0.05, and average signal-to noise A >64) is available in Additional data files 1-3. *Control with LB log cells; ^†^control with LB stationary phase cells; and ^‡^control with sessile cells from microcosm without plant. ^§^Genes passing the Bonferroni cutoff after a combined analysis of the nine microarrays altogether. A dash is used to mark those *rup *genes not passing the Bonferroni cutoff, although they did pass the Benjamini and Hochberg adjustment. LS, low signal (below cutoff).

The combined analysis of the three experiments as a group (nine microarrays) identified a larger number of genes induced and repressed in rhizospheric cells than when the independent analysis, followed by clustering, was done as described above. This was an expected statistical consequence of the increased number of tests in the analysis. Table 2 shows the numbers before and after applying corrections of the *P *value. Even with the strictest procedure, the Bonferroni correction [12], which is scarcely used in microarray studies due to its stringency, 57 genes appeared as up-regulated in *P. putida *in the rhizosphere. Of therse, 54 were part of the group of 90 mentioned above as a result of the independent analysis (Figure 2). No repressed gene passed the cutoff with the Bonferroni method (Additional data file 2). The remaining 36 up-regulated genes identified with the independent analysis were part of the group of over 300 obtained after applying a less strict correction [13] to the combined analysis (Figure 2; Additional data file 3). With this method, a substantial number of genes appear as rhizosphere-repressed, although the majority show fold changes close to the -2 cutoff (Additional data file 3).

**Table 2:** Number of differentially expressed genes in the rhizosphere

	Induced	Repressed
Non-adjusted *P *value	376	119
Benjamini and Hochberg	349	85
Bonferroni correction	57	-

Result of the combined analysis of nine microarrays, three biological replicates per experiment and three experiments (each with a different control). See the text for details. Dash indicates no gene passed the cutoff.

### Real time RT-PCR confirmation of changes in the mRNA levels of rhizosphere differentially expressed genes

To validate our microarray data by an independent technology, gene expression of six genes among those identified as rhizosphere up-regulated was examined in the rhizosphere versus microcosm by real time RT-PCR. Other neighbor genes not identified in the microarrays as rhizosphere induced were also included in the RT-PCR analysis, that is, PP1477 and PP4988, which likely form part of same transcriptional units as PP1476 and PP4987 (Table 1), respectively, and PP3744, the *glc *transcriptional activator of the rhizosphere induced gene encoding PP3746 (Table 1). All these genes were differentially expressed with a fold change higher than three (Table 3), confirming the microarray results, and indicating that if genes are listed in Table 1 it does not necessarily mean they were not induced in the rhizosphere. Restrictive conditions imposed to pass the cutoff might be the cause of this underestimation. Gene expression variability within the same operon may also occur, due to factors such as different mRNA stability or the existence of internal promoters.

**Table 3:** Differential gene expression of *rup *genes (real time RT-PCR)

Gene	Fold change^a^
PP1476 - conserved hypothetical protein	5.07 ± 0.4
PP1477 - *recJ*-single-stranded-DNA-specific exonuclease RecJ	5.48 ± 0.7
PP2076 - hypothetical protein	7.13 ± 0.4
PP2560 - transport protein HasD putative	3.84 ± 0.3
PP3744 - *glc *operon transcriptional activator	3.80 ± 0.2
PP3746 - glycolate oxidase, subunit GlcE	21.30 ± 1.5
PP4987 - chemotaxis protein putative	6.51 ± 0.9
PP4988 - chemotaxis protein putative	5.02 ± 0.8
PP5321 - *phoR*-sensory box histidine kinase PhoR	4.58 ± 0.1

^a^Rhizosphere versus microcosm. Average of three samples and standard deviation are shown.

### Reliable rhizosphere up-regulated (*rup*) genes

Following the initial premise of identifying genes with a reliable and specific change in their expression levels, we focused our attention primarily on the 93 genes showing increased expression in the rhizosphere (90 obtained from the independent analysis and the additional 3 that passed the Bonferroni adjustment of the combined analysis; Table 1) with respect to any other condition. About one-third of these encode hypothetical proteins whose specific functions have yet to be determined (Additional data file 1).

The remaining genes with increased expression in the rhizosphere provide an ample view of the determinants at play in this plant-bacterial interaction (Table 1), some confirming previous data about the participation of elements such as flagella or thiamine (vitamin B1) biosynthesis. One conclusion to be drawn is that aside from interspecific competition, which is not contemplated in these experiments, two opposing forces act simultaneously, driving bacterial adaptation to life in the rhizosphere. On one hand, nutrient availability is reflected by the increased expression of genes involved in the uptake of certain carbon and nitrogen sources (in particular amino acids, dipeptides and polyamines), some of them, like glycolate, excreted by plants, as well as metabolic and degradative functions (degradation of aromatic compounds such as phenylacetic and/or phenylalkanoic acids, sarcosine oxidase, plant exopolymers β-glucosidase, urease). The high induction of an urease accessory protein may be related to the limiting nitrogen source, since the nitrogen is likely being used by the plant. Alternatively, compounds other than urea in the root exudates of corn plants might be responsible for this induction, since urea is not produced by this plant [14]. On the other hand, genes coding for stress response and detoxification proteins also show increased expression in the rhizosphere. These include glutathione peroxidase, a protein of the Pmp3 family, or the fatty acid *cis-trans *isomerase Cti, which has been related to stress adaptive mechanisms, in particular to membrane-toxic organic compounds [15,16], as well as several putative efflux transporters of toxic compounds. This indicates the necessity to cope with oxidative stress and other damaging agents, for instance, secondary metabolites present in seed and root exudates that show antimicrobial activities [17,18]. In this context it has been shown that the TtgABC efflux pump of *P. putida *recognizes a wide set of flavonoids [19]. However, these results should also be interpreted from a second perspective, and not just as defense mechanisms required by rhizosphere colonizers in order to benefit from the nutrients released by the plant. Reactive oxygen species produced in root tissues have been implicated not only in stress but also in signaling processes in legume-rhizobia symbioses [20]. Such a potential role has recently started to be investigated in other mutualistic associations [21].

Plant-bacterial signaling may be reflected by another relevant group of genes induced in the rhizosphere, comprising signal transduction sensors and response regulators, as well as three transcriptional regulators of the AraC and TetR families. In this group is included the sensor histidine kinase PhoR, which participates in the global response to inorganic phosphate limitation. Autophosphorylation of *phoR *in *Bacillus *has been reported to be modulated by the redox state, so that terminal oxidases are required for the Pho system's full induction [22]. Interestingly, two genes predicted to be involved in aa_3_-type cytochrome c oxidase assembly (PP0109 and PP0110) are induced in the rhizosphere. In other microorganisms, the Pho regulon has been implicated, among other processes, in biofilm formation and includes genes for the synthesis of antibiotics and other secondary metabolites [23-25]. The importance of secondary metabolism in mutualistic plant-*Pseudomonas *interactions is being studied from the point of view of the plant [17] and of the microorganism [26]. In our study, PP3786, predicted to participate in the synthesis of an as yet undefined secondary metabolite, was induced in the rhizosphere.

Finally, it is worth noting the expression of genes pointing towards potential DNA transfer and rearrangements that may take place in the rhizosphere. These include a helicase and the insertion sequence *IS*Ppu14 transposase, of which the six copies present in the genome of KT2440 were induced in the rhizosphere (five at a significant level; Additional data file 3).

### Role of rhizosphere up-regulated genes in colonization fitness

**Figure 3:**
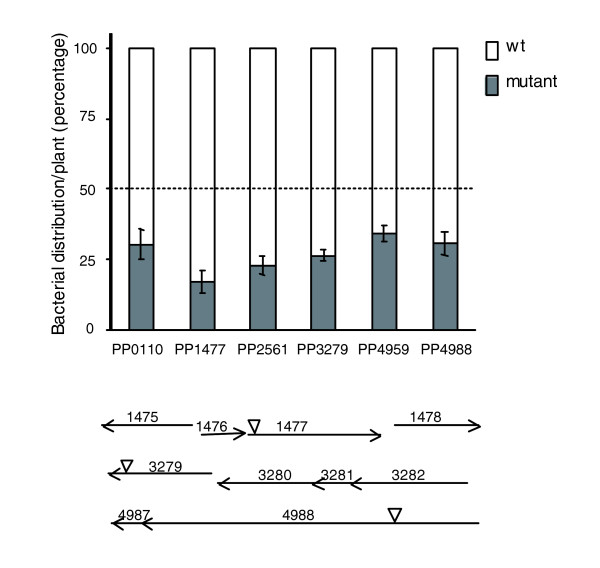
Rhizosphere fitness of mutant strains in competition with KT2440RTn*7*-Sm. The knocked-out open reading frame in the mutant strains is indicated by the locus name. Proportion of mutant (grey) and wild type (white), which was 50% ± 2% in the initial inocula, is plotted after 12 days of colonization. Data are the averages and standard error for six plants. KT2440RTn*7*-Sm, a streptomycin resistant derivative of KT2440R (see Materials and methods), was used as the wild-type strain in the experiments. KT2440RTn*7*-Sm and KT2440R are equally competitive in root colonization (not shown). Sm resistance marker of the wild-type bacteria allowed their specific selection against the mutants, which were kanamycin resistant derivatives of KT2440R. Statistical analysis was carried out using SPSS software (version 12.0.1 for Windows, SPSS Inc., Chicago, IL, USA). The linear model univariate analysis of variance rendered significant differences for the mutants shown in the figure (*P *value < 0.05) in comparison with the wild type. Seed adhesion rate was similar for mutants and KT2440R (0.5%), with the exception of PP0110 and PP1477 (0.1%). The growth of the mutants under laboratory conditions (rich and defined medium) was indistinguishable from that of KT2440RTn7-Sm. The transcriptional organization of mutated genes is shown in the bottom. The space between the 3' end of PP1476 and the 5' end of PP1477 is 7 bp. Translational coupling between PP3281 and PP3280 (8 bp) was observed. PP3279 is probably in an independent operon; however, PP3279 and PP3281 code for enzymes in the same degradative pathway [28]. Translational coupling between PP4987 and PP4988 was also observed (8 bp). Arrows indicate direction of transcription. Transposon insertion is indicated by inverted triangles.

To give an ecological significance to our results, we analyzed the role of several *rup *genes in competitive colonization and found that mutants in some *rup *genes are hampered in their survival in the rhizosphere in competition with the wild type, while they are indistinguishable from it under laboratory conditions. Nine transposon insertion mutants were chosen to test the relevance of rhizosphere expressed genes for the establishment of the *Zea mays*-*P. putida *association and microbial fitness in the rhizosphere. The mutants were selected to represent various classes of physiological roles (respiratory chain, transport, metabolism, stress adaptation, motility, transcriptional regulation and also hypothetical proteins). In some cases where the rhizosphere-induced gene is part of an operon, available mutants in genes included in the transcriptional unit were used. Competitive rhizosphere colonization assays were performed, and the proportion of each strain in the rhizosphere population was assessed after 12 days (Figure 3). In five cases, the wild type had displaced the mutant to a significant extent, so that the later represented less than 30% of the total population, supporting the idea that genes differentially expressed in the rhizosphere versus all the controls are important for bacterial fitness in this environment. The identification of KT2440 genetic determinants with a specific role in rhizosphere fitness constitutes a relevant result, since previously identified mutants of this strain hampered in colonization were also affected in growth under laboratory conditions [27] (our unpublished results). Most of the mutants identified here are also hampered in root colonization of the model plant *A. thaliana *(our unpublished results). Two of these mutants, PP0110 and PP1477, are also affected in adhesion to corn seeds (data not shown), indicating that their role is directly related to life on plant surfaces. The open reading frame encoding PP1477 is located 9 bp downstream to the *rup *gene encoding PP1476, so that polar mutations in PP1476 likely affect the expression of PP1477. The most noticeable result was obtained with mutant PP0906, which is affected in a putative multidrug efflux transporter, in agreement with the notion that the ability to cope with toxic compounds is one of the key traits for survival in the rhizosphere. However, this mutant was hampered in growth under laboratory conditions and was not further considered. Mutant PP3279 is affected in aromatic compound metabolism, specifically in CoA activation of phenylacetic acid [28], perhaps its reduced fitness being a consequence of its inability to remove toxic compounds from the exudates. Another mutant in the phenylacetyl-CoA pathway, PP3283, which forms part of the same functional unit as the *rup *gene ecoding PP3281 [28], was also affected in competitive colonization (not shown). The fifth mutant is affected in a type I secretion system of an exported protein with animal peroxidase and calcium binding domains. Two other mutants also showed reduced competitive colonization capacity, although to a lesser extent. These genes are nevertheless interesting. PP4959 codes for a response regulator containing a signal receiving domain and GGDEF/EAL domains, which have been implicated in regulating the transition from planktonic to sessile life styles through secondary messenger c-di-GMP levels [29]. Mutant PP4988 is affected in a sensor histidine kinase that forms part of a chemotaxis signal transduction operon (comprising loci PP4990 to PP4987), which in *P. aeruginosa *controls twitching motility mediated by type IV pili [30]. A role for type IV pili in biofilm formation as well as in attachment to legume roots has been reported [31,32], but this is the first indication that signal(s) present or absent in the root environment may trigger type IV pili functionality.

Transcriptional profiling *in vitro *[8] serves to pinpoint relevant components of exudates and how these influence bacterial physiology, but it obviates some of the conditions characteristic of the actual situation in the rhizosphere, in particular the association between bacterial cells and the plant root surface. A comparison of the data obtained here with that work shows limited overlap. Six genes identified in *P. aeruginosa *with increased expression in the presence of sugar beet exudates are homologs of *P. putida *genes induced in the corn rhizosphere, such as the helicase PP2565, or functionally related to them, like *soxB *(encoding sarcosine oxidase β-subunit). These are likely to reflect common characteristics of the root exudates of both plant species and/or compounds causing equivalent responses. With respect to genes previously identified in *P. putida *by *in vivo *expression technology, it is worth mentioning the PP1476/PP1477 operon, which as described above, is required for efficient colonization of seeds and roots. PP1476 encodes a homolog to *E. coli *YaeQ, which compensates for the loss of RfaH [33], a specialized transcription elongation protein. PP1477 corresponds to RecJ, an exonuclease involved in recombination and DNA repair after UV [34] or oxidative damage [35], again supporting the view of the rhizosphere as an environment where nutrient availability comes at an extensive cost in terms of the battery of protection mechanisms that have to be kept active. This work opens a challenging perspective to the study of mutualistic plant-microbe associations where, besides other determinants, energetic balances should be taken into account as part of the factors that define the success of these cross-kingdom interactions.

## Conclusion

**Figure 4:**
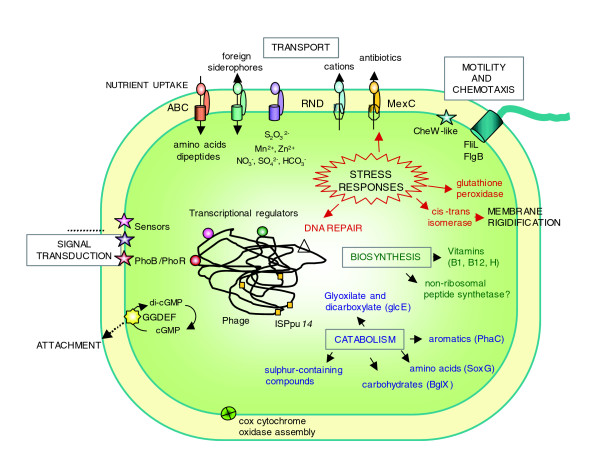
Integrated scheme showing relevant bacterial functions induced in the rhizosphere-*Pseudomonas *interaction. Functions related to genes included in Additional data file 3 have also been included in this figure. See text for details.

The current study constitutes, to our knowledge, the first report on bacterial genomics in the rhizosphere. The main functions identified in this transcriptional profiling study as being specifically expressed in the rhizosphere are integrated in the scheme shown in Figure 4. Future work should aim at unveiling the regulatory mechanisms that control such reprogramming of transport, metabolic and stress-related functions. We have also demonstrated that RNA samples of good quality and in enough quantity can be obtained from a bacterial population growing in this complex environment so that parameters of great interest in the plant-*Pseudomonas *interaction, such as the physical contact between the root and the bacteria and also the constant supply of root exudates, can be considered in gene expression studies. This work opens a challenging perspective to the study of mutualistic plant-microbe associations where, besides other determinants, energetic balances between nutrient availability and stress resistance should be considered to explain the success of these interkingdom relationships.

## Materials and methods

### Bacterial strains, culture conditions, and solutions

*P. putida *KT2440 is a derivative of *P. putida *mt-2, which was isolated from a vegetable-planted field [36]. The genome of KT2440 is completely sequenced [37]. Rifampin-resistant derivative KT2440R was generated elsewhere [38]. All the mutants used in competitive root colonization assays are derivatives of KT2440R and exhibit kanamycin resistance from the miniTn*5 *transposon insertion that causes the mutation. The transposon insertion site was determined by using arbitrary PCR at the *Pseudomonas *Reference Culture Collection [39] and is available upon request. KT2440RTn*7*-Sm was generated by site specific insertion of miniTn*7*-ΩSm1 at an extragenic site near *glmS *[40] in KT2440R. *P. putida *strains were routinely grown at 30°C in LB medium, except for microarray experiments, in which bacteria were cultivated at 24°C and 200 rpm. When appropriate, antibiotics were added to the media at the following concentrations (μg/ml): kanamycin, 25; streptomycin, 100; rifampin, 10.

### *P. putida *microarrays

The technical description of the *P. putida *genome array is available at the ArrayExpress repository for microarray data (accession number A-MEXP-949). This genome-wide DNA chip has been used elsewhere [41-43].

### Experimental set-up for preparation of the control samples for microarray experiments

Controls in microarray experiments consisted of: LB log phase cells (OD_660 nm _= 0.7); LB stationary phase cells (OD_660 nm _= 3.3); and early stationary phase bacteria from sand microcosms. Microcosms consisted of 50 ml Sterilin tubes containing 40 g of sterile water-washed silica sand. About 10^5 ^CFU was incubated at 24°C for 18 h and bacterial growth was supported with 4 ml of plant nutrient solution [7] supplemented with sodium citrate 15 mM, Fe-EDTA 100 μM, and micronutrients of Murashige and Skoog medium (MS) [44]. To recover cells from the microcosms, they were shaken at 400 rpm for 2 minutes with 80 ml M8 salts [45] and left standing for 15 s. All cells from bacterial suspensions were collected by centrifugation at 4°C (6,700 × g, 8 minutes) in tubes precooled in liquid nitrogen. Pellets were immediately frozen in liquid nitrogen and conserved at -80°C.

### Surface sterilization, germination of seeds and root colonization assay

Corn seeds were surface-sterilized by rinsing with sterile deionized water, washing for 10 minutes with 70% (vol/vol) ethanol, then for 15 minutes with 20% (vol/vol) bleach, and followed by thorough rinsing with sterile deionized water. Surface sterilized seeds were pregerminated on MS medium [44] containing 0.2% (wt/vol) phytagel (Sigma P8169, St. Louis, MO, USA) and 0.5% (wt/vol) glucose, which was used instead of sucrose to monitor any contamination of the seeds, at 30°C in the dark for 48 h. For root colonization assays, seeds were inoculated with approximately 5 × 10^6 ^CFU/ml from LB medium overnight culture suspended in M9 salt [45]. After incubation without shaking for 30 minutes at 30°C, seeds were washed and planted in 50 ml Sterilin tubes containing 40 g of sterile washed silica sand and 10% (vol/wt) plant nutrient solution supplemented with Fe-EDTA and MS micronutrients as described above, being the final inoculum size of 10^5 ^CFU. Inoculated plants were maintained in a controlled chamber at 24°C and 55-65% humidity with a daily light period of 16 h. After 6 days, plants were collected, shoots discarded and the roots placed in 50 ml Sterilin tubes containing 15 ml of M8 salts [45] and 4 g of glass beads (3 mm diameter). Tubes were vortexed for 2 minutes, left standing for 15 s and cells from bacterial suspensions collected by centrifugation for 8 minutes at 6,700 × g (4°C) in tubes precooled in liquid nitrogen. Pellets were immediately frozen in liquid nitrogen and conserved at -80°C.

### Competitive root colonization assays

Surface sterilization, germination of seeds, and bacterial inoculation were performed as described in the previous section, except seedlings were inoculated with a mix of KT2440RTn*7*-Sm, as the wild type, and the mutant strain in the specified *rup *gene. Inocula size differences between wild-type and mutant strains were less than 2%. At the indicated times, bacterial cells were recovered from the rhizosphere as specified above. LB-agar supplied with rifampin and streptomycin (or kanamycin) was used to select KT2440RTn*7*-Sm or the mutant strains, respectively.

### RNA purification and preparation of labeled cDNA

Total RNA from the bacteria recovered from the rhizosphere of six plants and from the control samples was extracted by using TRI Reagent (Ambion, ref. 9738, Austin, TX, USA) as recommended by the manufacturer except that Tripure Isolation reagent was preheated at 70°C followed by purification with RNeasy columns (Qiagen, cat no. 74104, Hilden, Germany). RNA concentration was determined spectrophotometrically and its integrity was assessed by agarose gel eletrophoresis. RNA (30 μg) was primed with 30 μg of pd(N)_6 _random hexamers (Amersham, cat. no. 27-2166-01, Piscataway, NJ, USA). Indirect cDNA labeling was used to generate fluorescent probes for hybridization. cDNA synthesis was performed at 42°C for 2 h in a 30 μl reaction volume containing 0.5 mM (each) dATP, dCTP, and dGTP; 0.25 mM (each) dTTP and aminoallyl-dUTP (aa-dUTP; Sigma cat. no. A0410); 10 mM DTT; 40 U of RNaseOUT (Invitrogen, ref. 10777-019, Carlsbad, CA, USA); and 400 U of SuperScript II reverse transcriptase (Invitrogen, ref. 18064-014) in reverse transcriptase reaction buffer. The reaction was stopped by adding 10 μl of 50 mM EDTA and the RNA template was hydrolyzed with the addition of 10 μl of 1 N NaOH followed by incubation at 65°C for 15 minutes. Samples were then neutralized by adding 25 μl of 1 M HEPES (pH 7.5) and the hydrolyzed RNA and residual dNTPs were removed using QIAquick PCR purification columns (Qiagen, ref. 28104) according to the manufacturer's recommendations, except that the Tris-based elution buffer supplied with the kit was replaced with phosphate elution buffer (4 mM KPO_4 _pH 8.5) to avoid interferences with the subsequent labeling. cDNA samples were dried in a Speed-Vac to completion. Dried aminoallyl-labeled cDNA was resuspended in 9 μl of 0.1 M sodium carbonate buffer (pH 9.0), mixed with either Cy3 (control) or Cy5 (rhizospheric samples) fluorescent dyes (mono-reactive NHS-esters; Amersham Biosciences, cat. no. PA23001 and PA25001, respectively), and allowed to couple for 2 h at room temperature in the dark. After coupling, the reaction was quenched with 4.5 μl of 4 M hydroxylamine for 15 minutes. Finally, labeled cDNA probes were again purified with QIAquick PCR purification columns. Labeling efficiency was assessed using a NanoDrop ND1000 spectrophotometer (NanoDrop Technologies, Inc., Wilmington, DE, USA).

### Microarray hybridization and data analysis

Prior to the hybridization process, the microarray slides were blocked by immersion into 5× SSC (1× SSC is 0.15 M NaCl; 15 mM sodium citrate, pH 7), 0.1% (wt/vol) SDS, 1% (wt/vol) bovine serum albumin for 1 h at 42°C. Then, the slides were washed by two successive immersions in MilliQ water at room temperature, followed by a final wash with isopropanol. The slides were spin-dried by centrifugation at 1,500 × g for 5 minutes, and used within the next hour. Equal amounts of Cy3- and Cy5-labeled cDNAs, one of them corresponding to the control and the other one to the problem to be analyzed, were mixed, dried in a Speed-Vac and reconstituted in 35 μl of hybridization buffer (5× SSC, 25% (vol/vol) formamide, 0.5% (wt/vol) SDS, 5 × Denhardt's solution, 5% (wt/vol) dextransulfate) preheated to 42°C. The labeled probe was denatured at 98°C for 3 minutes, applied onto the microarray slide and covered with a glass coverslip. The slide was then introduced in a humidified hybridization chamber (AHC ArrayIt Hybridization Cassette; Telechem International, Inc., Sunnyvale, CA, USA) and incubated for 18-20 h in a water bath at 42°C, preserved from light. Following hybridization, the microarrays were washed by gentle agitation in 2× SSC, 0.1% (wt/vol) SDS at 42°C for 5 minutes, followed by a 5 minute wash at room temperature in 1× SSC, two 5 minute washes in 0.2× SSC, and a final 5 minute wash in 0.1× SSC. Finally, the slides were spin-dried in a centrifuge at 1,500 × g for 5 minutes, and scanned on a GenePix 4100A scanner (Axon Instruments, Inc., Foster City, CA, USA). Images were acquired at 10 μm resolution, and the background-subtracted median spot intensities were determined using GenePix Pro 5.1 image analysis software (Axon Instruments, Inc.). Spots with anomalies were discarded and excluded from further analysis. Spot signal intensities were normalized by applying the Lowess intensity-dependent normalization method [46], and statistically analyzed using the Almazen System software (Alma Bioinformatics SL, Madrid, Spain). Three independent biological replicates were performed for each experiment. *P *values were calculated by Student's *t*-test. A particular open reading frame was considered differentially expressed if: the fold change was at least 2; the *P *value was lower than 0.05; and the average signal-to noise (A) was at least 64. In the combined analysis of the nine microarrays, *P *values were adjusted for multiple testing to control the false discovery rate [13] or the type I family wise error rate by applying the Bonferroni correction [12]. Microarray data have been deposited in the ArrayExpress database (E-MEXP-949).

### Real time RT-PCR

Total RNA (1 μg) treated with *Turbo DNA free *(Ambion, ref 1907) was retrotranscribed to cDNA with Superscript II reverse transcriptase using random hexamers as primers. The specific primer pairs used to amplify cDNA were: 5'-CAGACTATCGCTCGGCAC and 5'-CGGCTCATCGACATCCGAC for the gene encoding PP1476; 5'-CGATCACCGGCGCATCACTT and 5'GTCCTCGCACAGCAGGCATT for the gene encoding PP1477; 5'-CGCTTCATCCAGCCTTCC and 5'-CGAGACGATCTGGTCGTTG for the gene encoding PP2076; 5'-GGCCGACGTGCTGTTTCACT and 5'-AGCATGCCTAAGGTGGTGAC for the gene encoding PP2560; 5'-CATCATCGACACCGAACAG and 5'-CCAGCAGGTCGAACAGAG for the gene encoding PP3744; 5'-GTCAGCAATCGCTGCCAATC and 5'-AAGCGGTTATCGAGCGTGTC for the gene encoding PP3746; 5'-CTCGCGTGGTGGTGCTCAAT and 5'-GCGCCACATCCACGTAGTTC for the gene encoding PP4987; 5'-GCTATGTGCAGCGTTGGTTG and 5'-CGCCATTGCAGGTAGCATTC for the gene encoding PP4988 5'-GACGCGGTGATCATGCTCG and 5'-GTGGCGCACCAGATTGGTC for the gene encoding PP5321; 5'-AAAGCCTGATCCAGCCAT and 5'GAAATTCCACCACCCTCTACC for the gene encoding 16S rRNA. Real-time RT-PCR was performed using iCycler iQ (Bio-Rad, Hercules, CA, USA). Target cDNA from the experimental and reference samples were amplified in triplicate. Each 25 μl reaction contained 1 μl of a dilution of the target cDNA (1:10-1:10,000), 200 μM dNTPs, 200 nM each primer, 3 mM MgCl_2_, 2.5 μl SyBR Green (Molecular Probes, Eugene, OR, USA), and 1 U Platinum *Taq *DNA Polymerase (Invitrogen) in PCR buffer (20 mM Tris-HCl pH 8.4, 50 mM KCl). Samples were initially denatured by heating at 95°C for 10 minutes. A 40-cycle amplification and quantification program was then followed (95°C for 15 s, 60°C for 30 s, and 72°C for 20 s) with a single fluorescence measurement per cycle according to manufacturers' recommendations. A final extension cycle (72°C, 1 minute) was performed. PCR products were between 106 and 300 bp in length. To confirm the amplification of a single PCR product, a melting curve was obtained by slow heating from 60°C to 99.5°C at a rate of 0.5°C every 10 s, for 80 cycles, with continuous fluorescence scanning. Results were normalized relative to those obtained for 16S rRNA. Quantification was based on analysis of threshold cycle (Ct) value as described by Pfaffl [47].

## Abbreviations

MS medium, Murashige and Skoog medium.

## Authors' contributions

MAM performed research. MEU analyzed data and wrote the paper. JJR-H contributed new reagents/analytic tools. JLR contributed to writing. MIR-G designed research, analyzed data and wrote the paper.

## Additional data files

The following additional data are available with the online version of this paper. Additional data file 1 is a table listing rhizosphere differentially expressed genes after independent analysis and clustering. Additional data file 2 is a table listing Rhizosphere induced genes after combined analysis applying Bonferroni *P *value correction. Additional data file 3 is a table listing Rhizosphere differentially expressed genes after combined analysis applying Benjamini and Hochberg *P *value adjustment

## Supplementary Material

Additional data file 1 Rhizosphere differentially expressed genes after independent analysis and clustering. Click here for file

Additional data file 2 Rhizosphere induced genes after combined analysis applying Bonferroni *P *value correction. Click here for file

Additional data file 3 Rhizosphere differentially expressed genes after combined analysis applying Benjamini and Hochberg *P *value adjustment. Click here for file
